# Visible-mid infrared ultra-broadband and wide-angle metamaterial perfect absorber based on cermet films with nano-cone structure

**DOI:** 10.1515/nanoph-2023-0021

**Published:** 2023-03-09

**Authors:** Fan Yang, Rui-Hao Li, Shi-Long Tan, Jian-Wen Dong, Shao-Ji Jiang

**Affiliations:** School of Physics, State Key Laboratory of Optoelectronic Material and Technologies, Sun Yat-Sen University, Guangzhou 510275, P.R. China

**Keywords:** cermet, metamaterial, nano-cone, perfect absorber, ultra-broadband

## Abstract

Metamaterial absorbers over a broadband spectrum with high absorption, good angular tolerance, and easy configurations have essential importance for optical and optoelectronic devices. In this study, a hybrid metamaterial absorber comprising multilayered cermet thin films (multi-cermet) with tapered structure is designed and experimentally demonstrated. Combining optical interference of multi-cermet films and optical field localization of nano-cone structures, the average absorbance of both simulation and measurement are more than 98% in an ultrabroad bandwidth (300–3000 nm), and the proposed absorber shows a good angular tolerance as well. The composite process of two easy-operated and efficient methods, colloidal lithography, and magnetron sputtering, is employed for large-area fabrication. In addition, owing to flexible polyimide substrate, the proposed absorber also shows good bending and heating resistance, which reflects its potential in engineering application.

## 1 Introduction

Metamaterial absorbers (MAs) with efficient absorbing properties at the visible and infrared wavelengths are attracting increasing interest, due to their potential optical applications including solar collection systems [1–5], thermal emitters [6–9], infrared imaging [10, 11], sensors [12–15] and photodetectors [16–18]. In order to meet the increasing demand of modern devices, it is necessary to achieve perfect absorption in a wide spectral band and maintain high absorbance under arbitrary incident angles. Thus, how to design and fabricate a perfect broadband absorber in the desired band should be of great concern.

Conventional perfect absorbers composed of metal–dielectric–metal (MDM) multilayer structures in the vertical direction are commonplace. In general, the response of this type of cavity is always angle-dependent and narrowband [19–24]. By employing a high-loss metal material and adding an impedance-matching layer atop, the absorption bandwidth can be broadened to a certain extent [25–27]. What’s more, numerous nanostructures have been brought up to broaden the bandwidth further and reduce the sensitivity to incident angle, such as pyramids, nanopillar, and nanoholes [19, 28], [29], [30]. However, most of these designs can only cover a part of the visible or near infrared (NIR) spectrum and their absorption bandwidth is inherently restricted. In addition, the existing manufacturing methods to realize these designs, have difficulty in achieving a good balance between high precision, time-consuming and cost [31, 32]. Thus, designing wide-angle and ultrabroadband perfect absorbers compatible with the currently used cost-effective industrial production technology remains a major challenge.

Here, as an initial step, we propose a planar multilayer cermet thin film (multi-cermet) absorber which is made of Cu/Zr–ZrO_2_ (HMVF, high metal volume fraction)/Zr–ZrO_2_ (MMVF, middle metal volume fraction)/Zr–ZrO_2_ (LMVF, low metal volume fraction)/Al_2_O_3_. Based on the optical interference of multi-cermet films, the designed multi-cermet absorber demonstrates well-matched impedance from 350 to 2670 nm, which leads to a good absorption ( >90% ) in such a wide region. In the previous study, we have proved that nanostructure can improve the absorption performance of planar absorber efficiently [33]. Therefore, we transfer this multi-cermet absorber to tapered polyimide (PI) substrate. Based on the plasma near-field coupling and excited magnetic resonance of light trapping structures, the absorption bandwidth is broadened further and the average absorbance of the proposed absorber is more than 98.7% from 300 to 3000 nm (experiment result: 98.1%). The absorber also exhibits excellent angular redundancy and polarization insensitivity. More prominent is that the centimeter-scale (2 cm × 2 cm) absorber is efficiently fabricated using the colloidal lithography method and magnetron sputtering composite process. In addition, owing to the use of flexible PI substrate, the proposed absorber also shows good bending resistance and heat resistance, which reflects its potential engineering value.

## 2 Design and calculation

### 2.1 Design of planar multi-cermet absorber

To begin with, according to the law of energy conversation, the absorbance (*A*) can be calculated by the formula *A* = 1 − *R* − *T*, where *R* and *T* represent the reflectance and transmittance, respectively [33]. By designing a thick metal (Cu) as the bottom layer, the transmittance can be almost reduced to zero. Then, absorption can be maximized with the minimized reflection at the interface with air.

**Figure 1:**
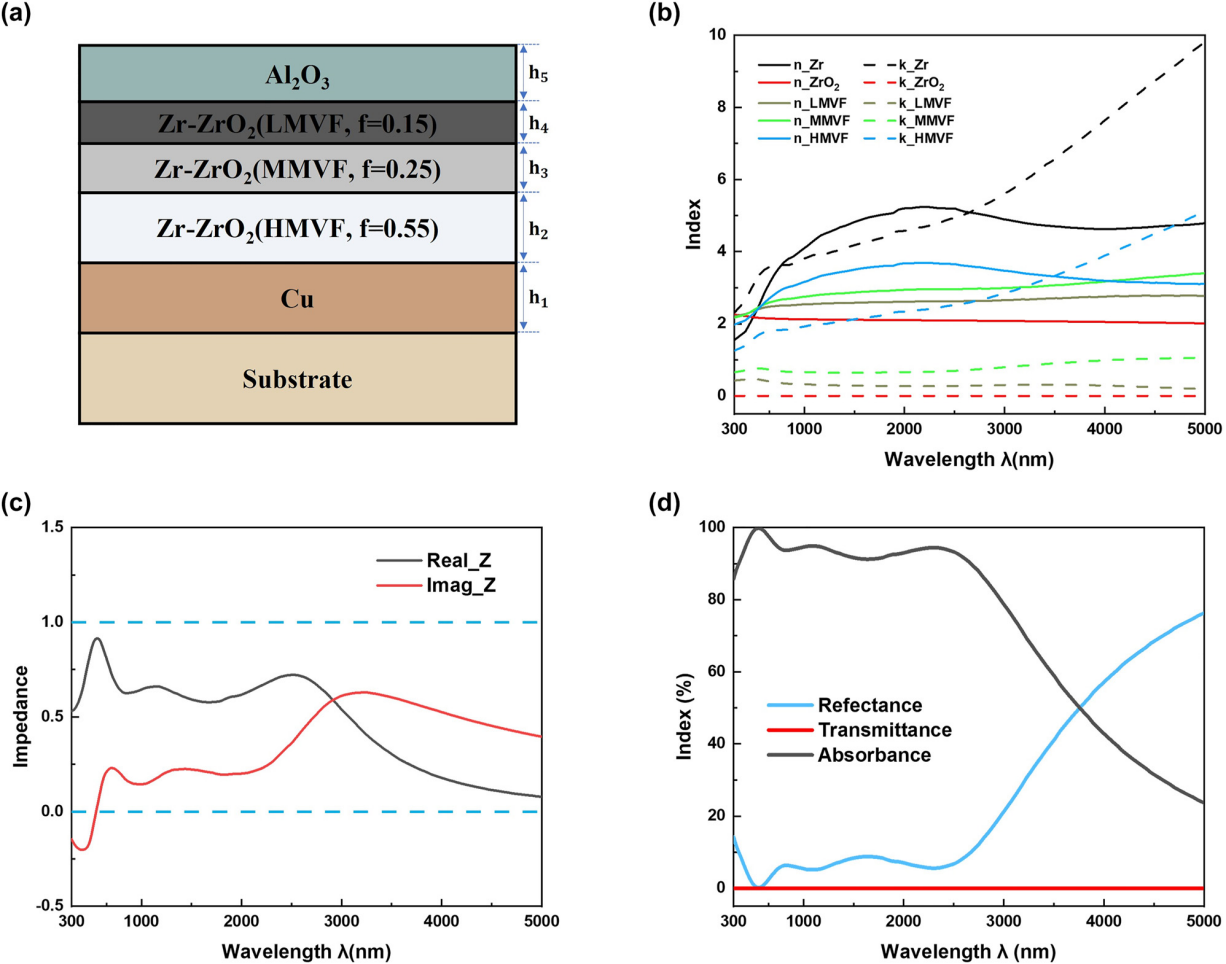
The simulation parameters and results of planar multi-cermet absorber. (a) The 2D schematic diagram of the planar multi-cermet absorber with structural parameters as h1 = 100 nm, h2 = 100 nm, h3 = 55 nm, h4 = 40 nm, h5 = 70 nm. (b) Refractive index *n* and extinction coefficient *k* of Zr–ZrO_2_ with different metal filling factor f. (c) Real (black line) and imaginary (red line) parts of the calculated impedance of the sample (the blue dotted lines represent the impedance of 0 and 1). (d) Simulated reflectance, transmittance, and the corresponding calculated absorbance of the proposed absorber at normal incidence.

To reduce interface reflection, three-layered cermet and a dielectric coating (Al_2_O_3_) stacked in the vertical direction are designed. The order of cermet layers from bottom to top is HMVF, MMVF, and LMVF. Here, lower metal volume fraction leads to lower effective permittivity (*ɛ*_eff_) value of the cermet. Consequently, the proposed multi-cermet absorber has a gradually increasing *ɛ*_eff_ from the upper air–structure interface to the lower structure–substrate interface which contributes to the impedance matching. The metal filling factor *f* is, respectively, set as *f* = 0.55 (HMVF), *f* = 0.25 (MMVF) and *f* = 0.15 (LMVF), considering the fabrication parameter. In addition, the test results of these metal filling factors were measured by energy dispersive spectroscopy (EDS) as shown in Table 1. Therefore, the planar multi-cermet absorber made of Cu/Zr–ZrO_2_ (HMVF, *f* = 0.55)/Zr–ZrO_2_ (MMVF, *f* = 0.25)/Zr–ZrO_2_ (LMVF, *f* = 0.15)/Al_2_O_3_ is designed as shown in Figure 1(a).

**Table 1:** The results of EDS for cermet coatings.

Materials	Atomic (%)		Metal filling factor *f*
	Zr	O	
Zr–ZrO_2_ (HMVF)	52.81	47.19	0.55
Zr–ZrO_2_ (MMVF)	40.15	59.85	0.25
Zr–ZrO_2_ (LMVF)	37.08	62.92	0.15

To guide the design of multilayer-cermet film system, the equivalent optical parameters of cermet can be calculated quantitatively by effective-medium-theory (EMT) method. The EMT method is based on a model proposed by Ping Sheng (SH) which has been widely used for the dielectric function of a composite [34, 35]. In the SH theory, a probabilistic growth model for grains in a composite film has been introduced. The film is modeled as a mixture of two types of coated oblate spheroidal units, dielectric-coated metal spheroids described as type-a units and metal-coated dielectric spheroids described as type-b units [36]. For the simple case of spherical grains, the relative probability of the occurrence for type-a units *J*_*a*_ at any metal volume fraction is given by [34, 36]

(1) $$J_{a}=\frac{{\left(1-f_{a}^{1/3}\right)}^{3}}{{\left(1-f_{a}^{1/3}\right)}^{3}+{\left[1-{\left(1-f_{a}\right)}^{1/3}\right]}^{3}}$$

For type-b units, *J*_*b*_ = 1 − *J*_*a*_. In the simple case of spherical grains, the average dielectric function of a composite in Sheng’s approximation, *ɛ* ^*SH*^ is given by [35]

(2) $$J_{b}\frac{\varepsilon_{1-}\varepsilon^{SH}}{\varepsilon_{1}+2\varepsilon^{SH}}+\left(1-J_{b}\right)\frac{\varepsilon_{2}-\varepsilon^{SH}}{\varepsilon_{2}+2\varepsilon^{SH}}=0\;$$

where

(3) $$\varepsilon_{1}=\varepsilon_{a}\frac{\left(2\varepsilon_{a}+\varepsilon_{b}\right)-2\left(1-f_{a}\right)\left(\varepsilon_{a}-\varepsilon_{b}\right)}{\left(2\varepsilon_{a}+\varepsilon_{b}\right)+\left(1-f_{a}\right)\left(\varepsilon_{a}-\varepsilon_{b}\right)}$$

(4) $$\varepsilon_{2}=\varepsilon_{b}\frac{\left(\varepsilon_{a}+2\varepsilon_{b}\right)+2f_{a}\left(\varepsilon_{a}-\varepsilon_{b}\right)}{\left(\varepsilon_{a}+2\varepsilon_{b}\right)-f_{a}\left(\varepsilon_{a}-\varepsilon_{b}\right)}$$

Here, the *ɛ*_*a*_ and *ɛ*_*b*_ are the dielectric functions of type-a units and type-b units, respectively. The filling factor *f*_*a*_ represents the volume fraction occupied by the type-a units. And the complex refractive index *n* + *ik*, is derived from the complex dielectric function *ɛ* = *ɛ*′ + *iɛ*″ using the relationship *n* + *ik* = *ɛ* ^1/2^. By using EMT, the refractive index *n* and extinction coefficient *k* of cermet could be calculated. Figure 1(b) presents the *n* and *k* of Zr–ZrO_2_ cermet with different metal filling factor. In the calculation, the optical constants of Zr are obtained from Ref. [37], the optical constants of ZrO_2_ are obtained from Ref. [38].

To reveal the absorption mechanism, we calculated the optical impedance *Z* of the multi-cermet absorber. In impedance theory, reflectance of absorber can be calculated by the formula, $R={\left|\left(Z-1\right)/\left(Z+1\right)\right|}^{2}$ [19, 39]. When the real and imaginary parts of *Z* are close to 1 and 0, respectively, the absorber shows a superior absorption. We extracted the effective optical impedance of the designed structure and depicted in Figure 1(c). Based on the optical interference of multi-cermet films, the designed absorber demonstrates well-matched impedance from 350 to 2670 nm, which leads to a good absorption in this region. Figure 1(d) depicts the simulated reflectance, transmittance, and the calculated absorbance of the proposed multi-cermet absorber at normally incidence. The planar absorber has good absorbance up to 90% at 350–2670 nm, and the absorption bandwidth is obviously broader than the conventional planar MDM absorber as represented in Figure S2.

### 2.2 Design of nano-cone metamaterial absorber

The planar multi-cermet absorber has already shown a good absorbability at a broadband region. However, it is sensitive to the incident angle and the absorption performance is limited. To tackle these drawbacks, we designed a nano-cone metamaterial absorber based on the planar one.

**Figure 2:**
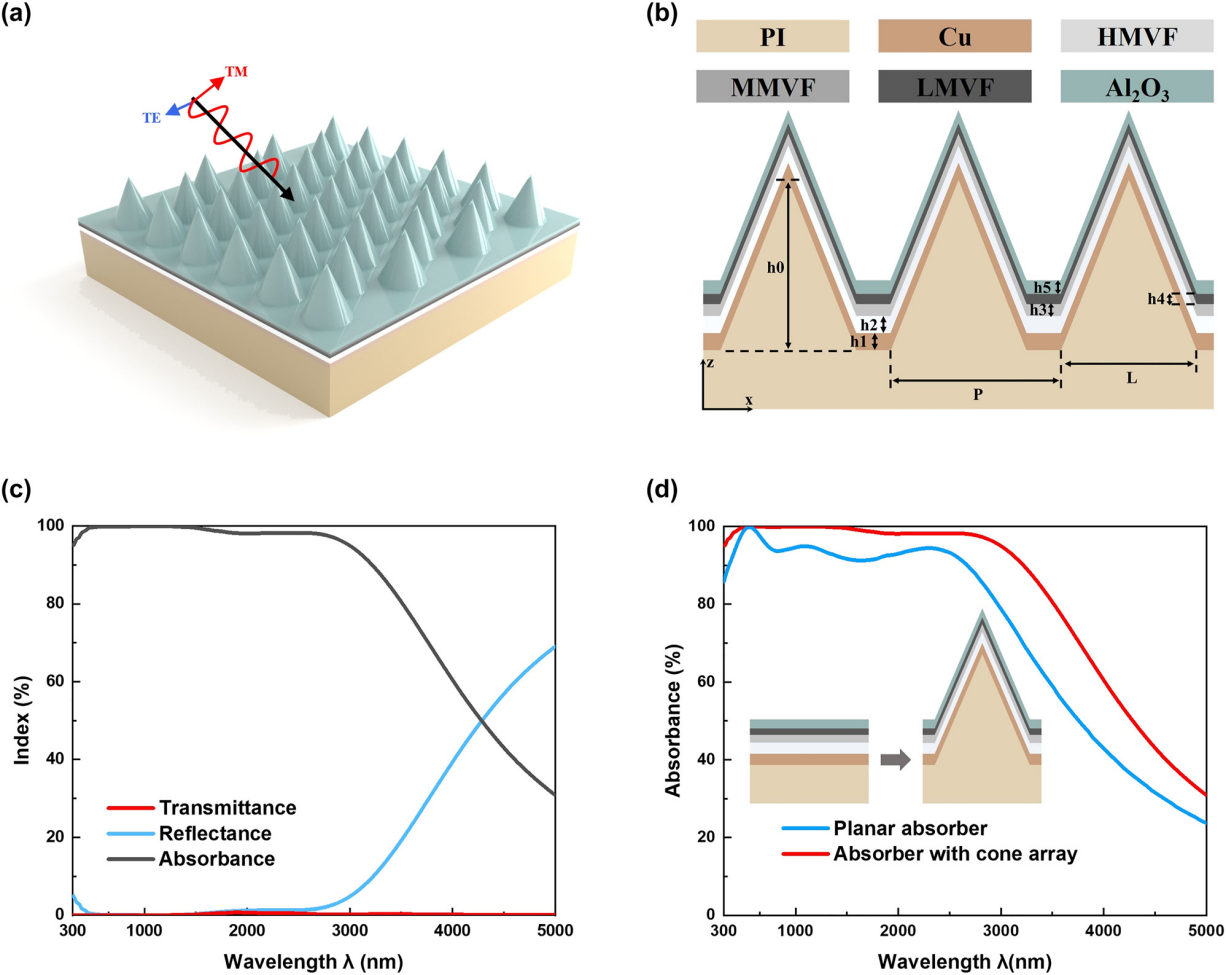
The simulation parameters and results of the designed nano-cone metamaterial absorber. (a) The 3D schematic diagram of the proposed nano-cone metamaterial absorber. (b) The cross-section configuration and structural parameters of the structure. (c) Simulated reflectance, transmittance, and the corresponding calculated absorbance of the proposed absorber at normal incidence. (d) Comparison of the calculated absorbance of the proposed nano-cone metamaterial absorber and the planar one.

The proposed metamaterial absorbers were composed of multi-cermet films with nano-cone structure as shown in Figure 2(a) and (b) presents the cross-sectional view. The period (*P*), height (h0), and bottom diameters (*L*) of the cone are set as 1000 nm, 1000 nm, and 800 nm, respectively, and the thickness of the five-layered thin film from bottom to top (h1, h2, h3, h4, h5) is set as 100 nm, 100 nm, 55 nm, 40 nm, and 70 nm, respectively.

The proposed absorber shown in Figure 2(a) and (b) is set up and systematically calculated by FDTD Solutions from LUMERICAL. The optical constant of Cu comes from Lorentz–Drude model with an experimental table data [40], the refractive index of Al_2_O_3_ is obtained from Ref. [41], the permittivity of PI is obtained from Refs. [42, 43] and the optical constant of Zr–ZrO_2_(*f* = 0.15, 0.25, 0.55) are obtained from the computed result as Figure 1(b) shows. Figure 2(c) depicts the simulated reflectance, transmittance, and the calculated absorbance of the proposed absorber when TM-polarized light is normally incident. The simulated transmittance is close to zero since the bottom Cu layer is much thicker than the penetration depth. Because of the additional light trapping structures, the proposed nano-cone metamaterial absorber presents better absorption performance than the planar one as shown in Figure 2(d). More specifically, it exhibits a high absorbance, greater than 95% in the range of 300–3000 nm, and the average absorbance is 98.7% in such a wide region.

**Figure 3:**
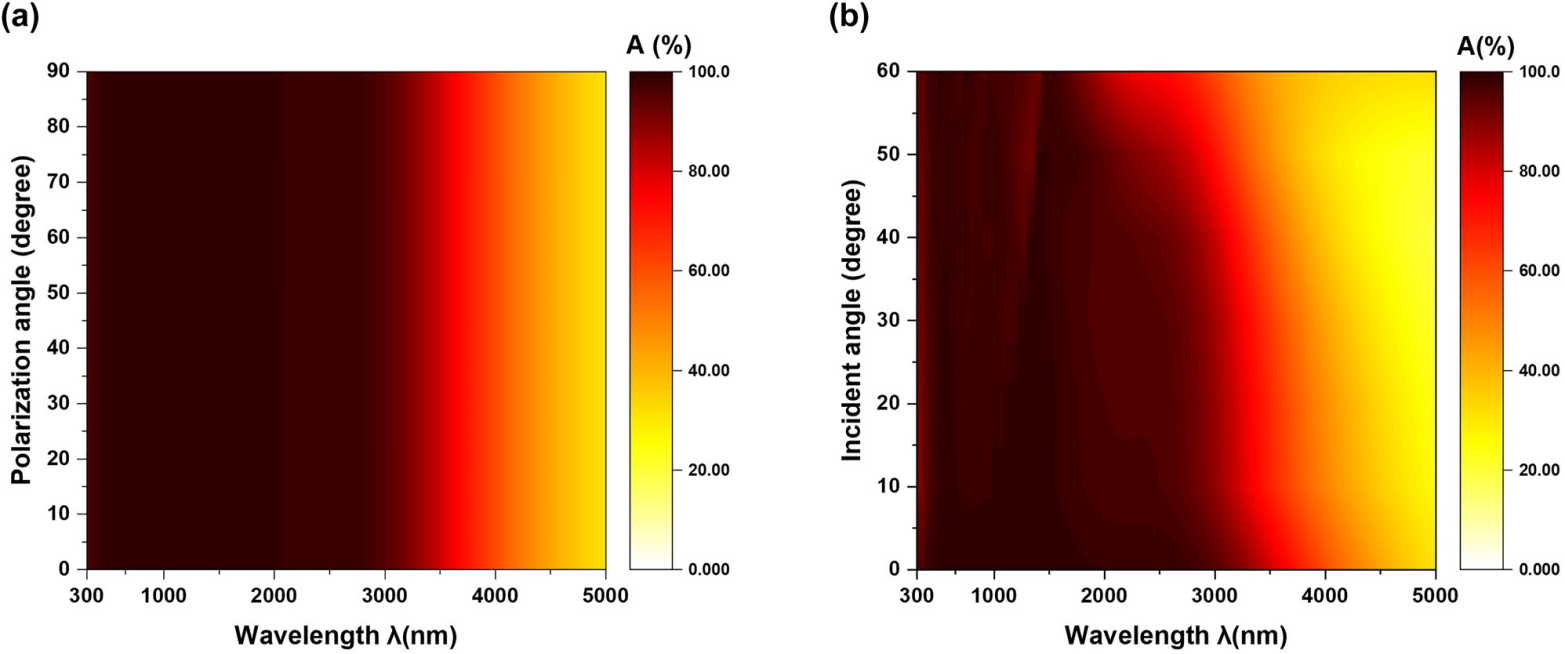
The simulated absorbance for different polarization angles and incident angles. (a) Calculated absorbance for different polarization angles from 0° to 90° at normal incidence. (b) Simulated absorption spectrum at different incident angles varying from 0° to 60° in 10° steps for unpolarized light.

Furthermore, the absorbance of different polarization states is calculated at normal incidence as shown in Figure 3(a). There is almost no difference between them due to the rotational symmetry of the designed structure. This result verifies the polarization-independence of the proposed absorber. The absorption spectrum is calculated for unpolarized light $\left[A_{\text{unpolarized}}=\left(A_{TE}+A_{TM}\right)/2\right]$ at different oblique incident angles, varying from 0° to 60° in 10° steps to determine the absorption performances related to incident angle. The range of absorbance exceeding 90% narrows slightly with the increase in the incident angle, as shown in Figure 3(b). Additionally, the average absorbance from 300 nm to 2900 nm still remains 90% at an incident angle of 60°, which indicates that the proposed absorber shows good angular tolerance while changing the incident angle.

**Figure 4:**
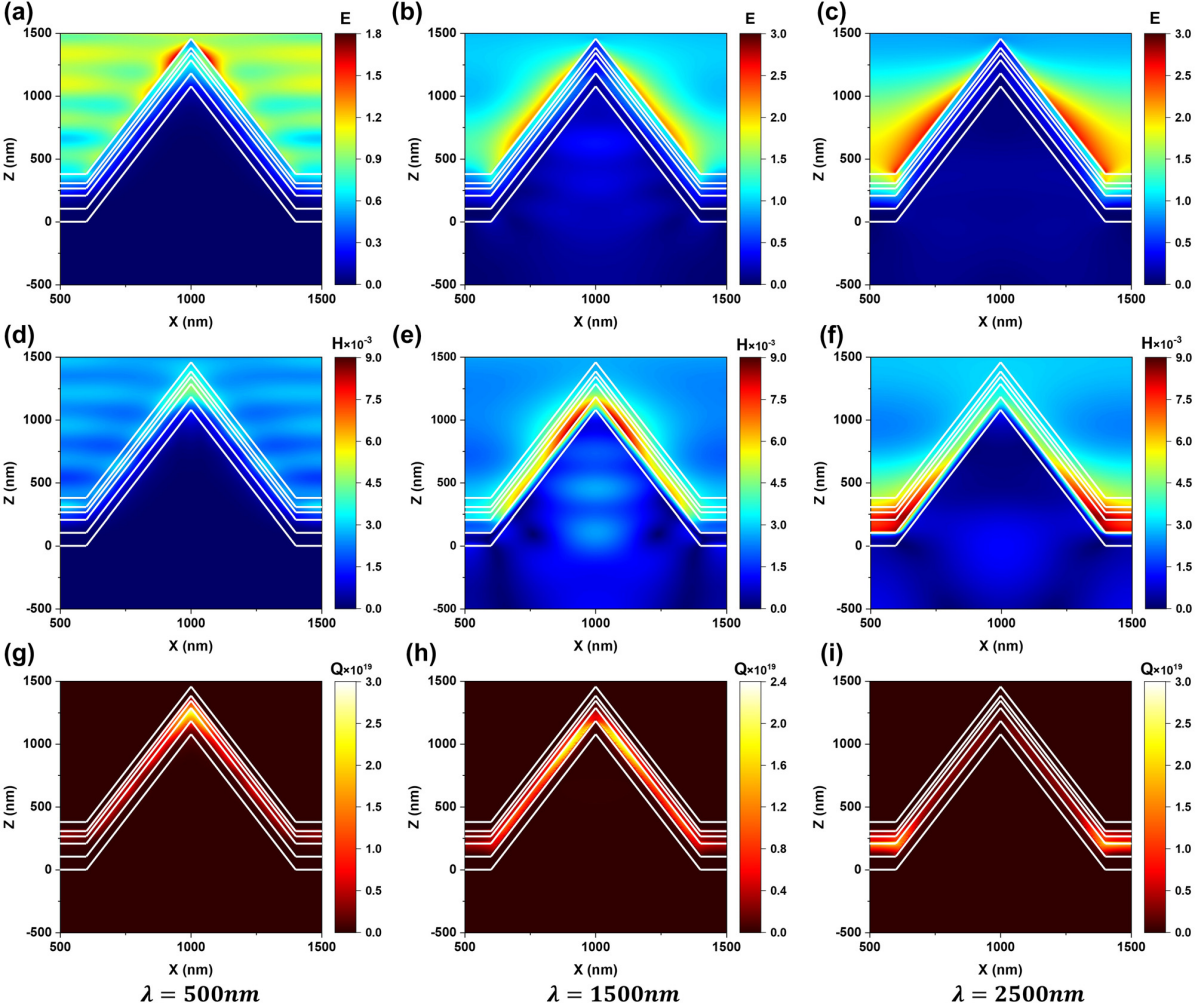
The contour plots represent the electric field intensity (a, b, c), magnetic field intensity (d, e, f) and the heat power density (g, h, i) at normal incidence for TM-polarized light with different incident wavelength.

We then simulated the electric field, magnetic field and heat power density distributions in our absorber at the specific incident wavelengths of 500 nm, 1500 nm, and 2500 nm, to further illustrate the underlying mechanism of the nano-cone light trapping structure as shown in Figure 4. The heat power density, *Q* is expressed as $Q=1/2\varepsilon_{0}\omega \varepsilon^{\prime \prime }{\left|E\right|}^{2}$ , where *ɛ*_0_, *ω*, *ɛ*″, and *E* represent the vacuum permittivity, angular frequency of the electromagnetic field, imaginary part of the permittivity and local electric field, respectively.

For short wavelength around *λ* = 500 nm, it can be seen from Figure 4(a) that the top of the cone presents a strong electric field, which means that the plasma near-field coupling between the metal arrays is excited [44]. Therefore, most of the energy is dissipated at the top of the proposed absorber, as shown in Figure 4(g). At medium wavelength around *λ* = 1500 nm, the enhanced magnetic field is localized on the sidewall of the absorber as shown in Figure 4(e), which can be attributed to the excitation of magnetic polaritons (MPs). The MPs lead to high absorption of the energy by the sidewall as shown in Figure 4(h). Here, an inductor–capacitor (LC) circuit can be used to analyze MPs and the resonance wavelength can be predicted by $\lambda =2\pi c_{0}\sqrt{LC}$ , where the inductive elements *L* and the capacitive elements *C* are related to the metal layer and the dielectric part [45]. The capacitance increases with the decrease in the gap between the structural units [30]. Therefore, the magnetic resonance generated by the electric displacement loop slowly moves to the bottom of absorber with the increase in the incident wavelength. As the result, at long wavelength around *λ* = 2500 nm, the enhanced magnetic field is localized at the bottom layer of the absorber as shown in Figure 4(f). Thus, most of the energy is dissipated at the bottom of the proposed absorber, as shown in Figure 4(i).

## 3 Fabrication

The predominant nanofabrication technologies are usually time-consuming and costly which result in the inability to efficiently fabricate nanostructures in large-area. To overcome the aforementioned drawbacks, a composite process of two easy-operated and efficient methods, colloidal lithography and magnetron sputtering, is integrated to obtain the proposed absorber.

**Figure 5:**
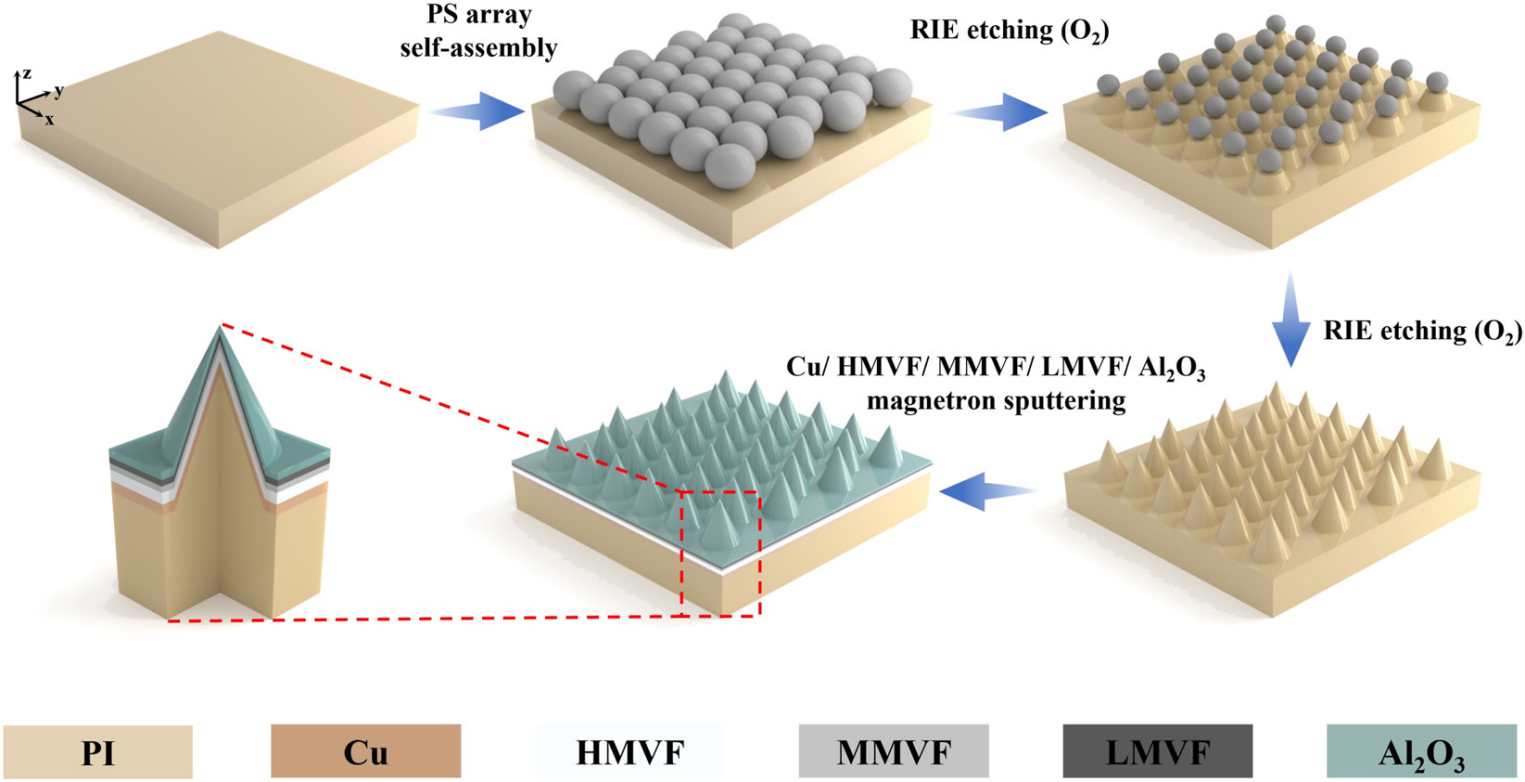
Schematic of the steps involved in the fabrication.

The fabrication process employs the microspheres self-assembly technology, reactive ion etching (RIE-501, Jingshengweina Technology), and magnetron sputtering (MSP-300, Jingshengweina Technology), as illustrated in Figure 5. Firstly, the PI substrate is ultrasonically cleaned in ethanol for 20 min to enhance its hydrophilicity. The 10%wt polystyrene (PS) microspheres (1 μm) aqueous solution and equal volume ethanol mixture is then added to deionized water mixed with 50 μL 10%wt sodium dodecyl sulfate (SDS). The PS microspheres are self-assembled into a large-area and hexagonal close-packed monolayer on the water–air interface due to the surface tension and capillary force. This monolayer is transferred to the prepared PI substrate after 24 h of drying in the air.

Secondly, with O_2_ RIE, both PS microspheres and PI substrate are etched effectively. The RIE are performed under a pressure of 2 Pa, an O_2_ gas flow rate of 20 SCCM and RF power of 50 W; the time of processes is set as 1500 s. So far, the tapered PI substrate is successfully prepared. Lastly, the five-layer thin film is deposited on the tapered PI substrate through the magnetron sputtering process and Table 2 presents the detailed parameters of the process. In detail, the metal filling factor f can be flexibly controlled by changing the Ar and O_2_ gas flow as well as the sputtering power. Consequently, a large-area (2 cm × 2 cm) absorber is efficiently fabricated.

**Table 2:** Parameters of magnetron sputtering.

Materials	Sputtering target	Gas flow/SCCM		Power	Time
		Ar	O_2_		
Cu	Cu	100	–	DC: 150 W	100 s
Zr–ZrO_2_ (HMVF)	Zr	60	9	RF: 610 W	107 s
Zr–ZrO_2_ (MMVF)	Zr	100	10	RF: 600 W	170 s
Zr–ZrO_2_ (LMVF)	Zr	100	10	RF: 575 W	85 s
Al_2_O_3_	Al_2_O_3_	100	–	RF: 400 W	800 s

**Figure 6:**
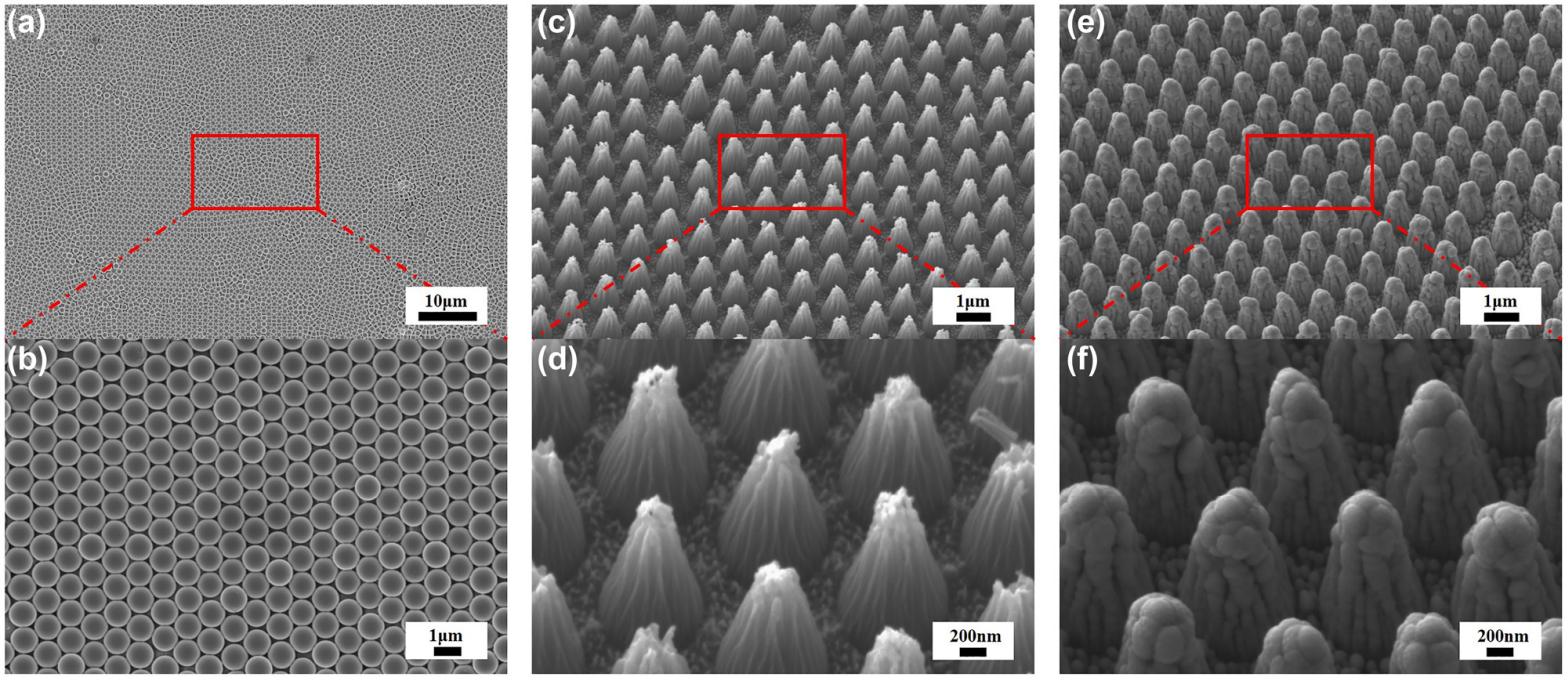
SEM diagram of the fabrication process. (a–b) SEM images of monolayer hexagonal PS microsphere arrays at 0° with different scale. (c–d) SEM images of tapered PI substrate at 30° with different scale. (e–f) SEM images of the proposed nano-cone absorber at 30° with different scale.

The scanning electron microscope (SEM) images of the prepared samples are characterized by the focused ion beam-scanning electron microscope (FIB-SEM, Auriga-4523, Zeiss). An ultrathin Pt layer is deposited on the surface to improve the conductivity of the samples. PS microspheres with a diameter of 1 μm formed a hexagonally close-packed monolayer on the 2 cm × 2 cm PI substrate, which act as a mask in the subsequent etching process, as represented in Figure 6(a) and (b). Figure 6(c) and (d) depict the prepared tapered PI substrate after the O_2_ RIE and ultrasonic cleaning processes. The height and the bottom diameters of the cones are approximately 1000 nm and 800 nm, respectively. The parameters of the cones can also be adjusted by the etching parameters. Then, five-layer cermet film is uniformly deposited on the sample, presenting a negligible change in the morphology of the sample, as shown in Figure 6(e) and (f).

## 4 Experimental results

The transmittance is close to zero since the thickness of the bottom copper layer is much greater than the penetration depth. Therefore, the measured absorbance is calculated as *A* = 1 − *R*. The absorption performance of sample is tested by measuring the reflection spectrum using a spectrophotometer (UV-3101 PC, Shimadzu) and absorption performance of the oblique incidence is tested from 10° to 60° using UV-VIS-NIR spectrophotometer (Lambda950, Wavetest) equipped with the absolute specular reflectance accessory.

**Figure 7:**
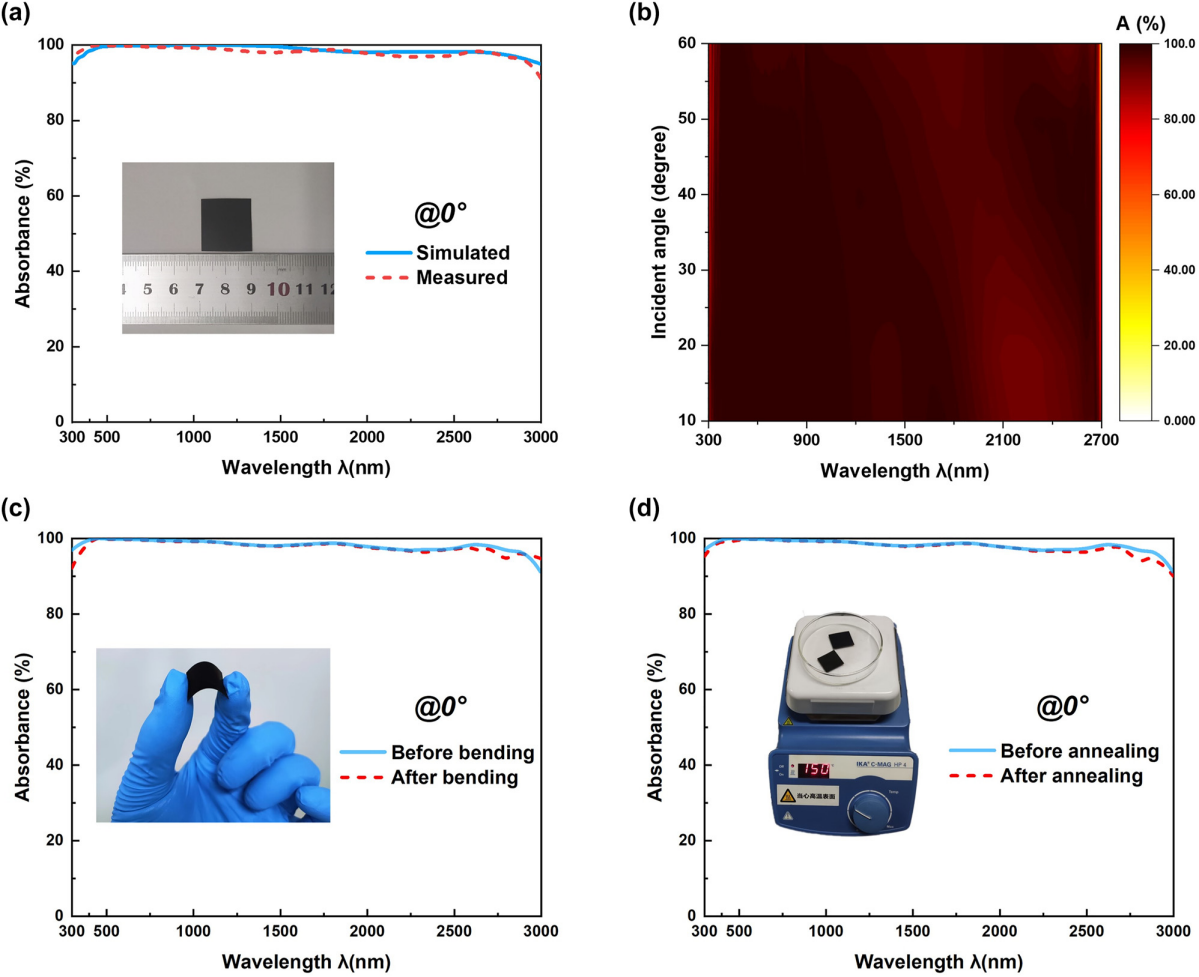
Test results of the sample's absorption performance. (a) The comparison between calculated and measured absorbance at normal incident angle for unpolarized light. (b) Measured absorption spectrum of prepared sample at different incident angles varying from 10° to 60° in 10° steps for unpolarized light. (c) Measured absorption spectrum before and after 100 times of bending tests at normal incident angle for unpolarized light. (d) Measured absorption spectrum before and after 6 h of 150 °C annealing tests at normal incident angle for unpolarized light.

Figure 7(a) presents the comparison between the simulated and measured absorbance from 300 nm to 3000 nm at normal incidence (0°) for unpolarized light and the inset is a photograph of the prepared centimeter-scale (2 cm × 2 cm) sample which appears black, indicating that it has good absorption performance in the visible regime. Figure 7(b) presents the measured absorbance at different oblique incident angles varying from 10° to 60° in steps of 10°. It can be concluded that the measured results correspond well with the simulated results, based on Figure 7(a) and the comparison between Figures 3(b) and 7(b). Surprisingly, the measured absorbance the measured absorbance shows better angular tolerance than the simulated results. This is mainly due to the presence of some metal nanoparticles on the sample surface during magnetron sputtering, which excites the localized surface plasmon [46, 47].

Additionally, due to the flexible PI substrate which has tolerance to high temperature, the prepared absorber shows good bending resistance and heat resistance. Additionally, the absorption spectrum of the prepared sample is compared before and after the 100 iterations of bending tests and the inset is the photography of the bending test, as shown in Figure 7(c). Furthermore, by using hot plates (C-MAG HP 4, IKA) the prepared absorber was heated to 150 °C for 6 h. And the absorption spectrum of the prepared sample is compared before and after annealing test and the inset is the photography of the annealing test, as represented in Figure 7(d). There is almost no difference in the absorption spectrum at normal incidence as Figure 7(c) and (d) reveals, indicating that the prepared absorber has great bending resistance and heat resistance.

## 5 Conclusions

In summary, this work proposes a design of broadband planar multi-cermet absorber made of Cu/Zr–ZrO_2_ (HMVF)/Zr–ZrO_2_ (MMVF)/Zr–ZrO_2_ (LMVF)/Al_2_O_3_. Then we present a simple yet generalized design for ultra-broadband metamaterial absorber which improves the absorption performance greatly, based on the planar one. By using the colloidal lithography method and magnetron sputtering composite process the centimeter-scale (2 cm × 2 cm) absorber is efficiently fabricated. The proposed metamaterial absorber is composed of polyimide (PI) substrate with nano-cone array and multi-cermet films. Based on the optical interference of the multi-cermet films, plasma near-field coupling and excited magnetic resonance of light trapping structures, the average absorbance of the proposed absorber is more than 98.7% (experiment result: 98.1%) from 300 to 3000 nm. Using spectrophotometer to test the fabricated absorber, it can be observed that the measured results correspond well with the simulated results and exhibit better angular tolerance. Additionally, owing to the use of flexible PI substrate, the proposed absorber also shows good bending resistance and heat resistance, which reflects its potential engineering value.

## Supplementary Material

Supplementary Material Details
