# Supplementary material for: Visible-mid infrared ultra-broadband and wide-angle metamaterial perfect absorber based on cermet films with nano-cone structure
Source: Nanophotonics. 2023 Mar 9;12(13):2451–60. doi: 10.1515/nanoph-2023-0021 (PMC11502049; doi:10.1515/nanoph-2023-0021)
Supplement: Supplementary file 1 — Supplementary Material Details [file j_nanoph-2023-0021_suppl_001.pdf]

Supplementary Material

Visible-mid infrared ultra-broadband and wide-angle metamaterial perfect absorber based on cermet films with nano-cone structure

Fan Yang, Ruihao Li, Shilong Tan, Jianwen Dong and Shaoji Jiang**

School of physics, State Key Laboratory of Optoelectronic Material and Technologies, Sun Yat-sen University, Guangzhou 510275, People's Republic of China

*dongjwen@mail.sysu.edu.cn; *stsjsj@mail.sysu.edu.cn

1 Calculation method of optical impedance

The impedance of the planar multi-cermet absorber is derived from the relationship $Z = 1/Y$, where Y represents the optical admittance. The optical admittance is given by [1]

$$Y = \frac{C}{B} \tag{1}$$

$$\begin{pmatrix} B \\ C \end{pmatrix} = \prod_{i=1}^{m} \begin{pmatrix} cos\delta_i & jsin\delta_i/N_i \\ jN_i sin\delta_i & cos\delta_i \end{pmatrix} \begin{pmatrix} 1 \\ N_{Substrate} \end{pmatrix} \tag{2}$$

Where $N_i = n + ik$ represents the complex refractive index of materials, i represents the number of each layer from top to bottom, and δ_i is given by

$$\delta_i = \frac{2\pi}{\lambda} \cdot N_i \cdot d_i \tag{3}$$

Here d_i represents the thickness of each layer. Therefore, both optical admittance Y and optical impedance Z can be quantitatively extracted as illustrated in Fig.S1 and Fig.1(c), respectively.

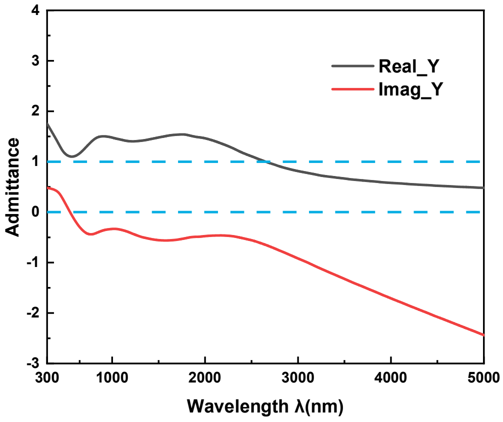

Fig. S1: Real (black line) and imaginary (red line) parts of the calculated optical admittance Y of the sample (the blue dotted lines represent the admittance of 0 and 1)

2 Comparison of multi-cermet absorber and the conventional MDM absorber

It is seen from Fig. S2 that the absorption bandwidth of multi-cermet absorber is obviously broader than the conventional MDM absorber. In addition, the structural parameters of MDM type absorber are set as Cr-SiO2-Cr-SiO2 (100nm-85nm-8nm-70nm).

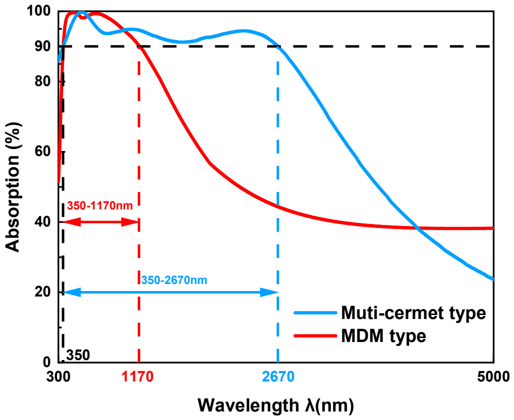

Fig. S2: Comparison of the calculated absorbance of the proposed planar multi-cermet absorber and the conventional planar MDM absorber.

3 Discussion with different structural parameters of nano-cone

The absorbance of proposed nano-cone metamaterial absorber with different bottom diameter L and height h0 are calculated as shown in Fig. S3. Keeping the other parameters constant, the absorption performance increases with the increase of L and h0. However, the larger L and h0, the more difficult to fabricate. Balancing the absorption performance and fabrication difficulty, the bottom diameter is set as L=800nm and the height is set as h0=1000nm.

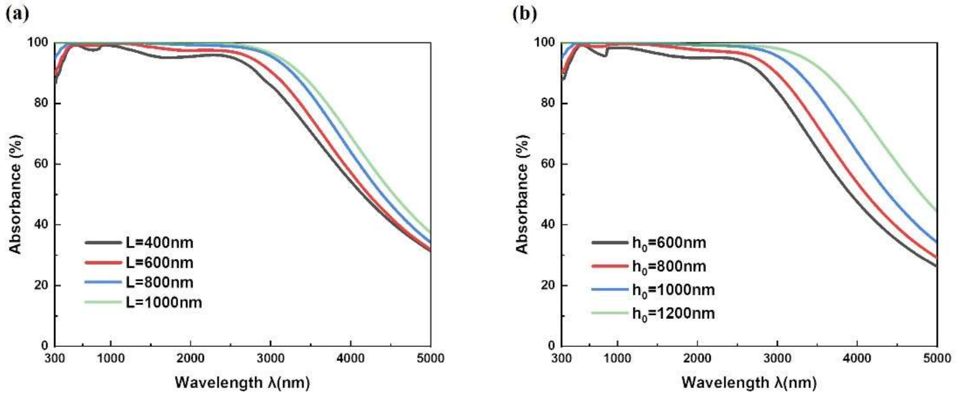

Fig. S3: (a) Calculated absorbance of the proposed nano-cone metamaterial absorber with different bottom diameters L. (b) Calculated absorbance of the proposed nano-cone metamaterial absorber with different height h0. Other parameters P=1000nm, h1=100nm, h2=100nm, h3=55nm, h4=40nm, h5=70nm.

4 Comparison of angular tolerances between the metamaterial absorber and the planar one

It is represented in Fig.S4 that the nano-cone metamaterial absorber shows better absorption performance and is less sensitive to incident angle, which shows its advantage.

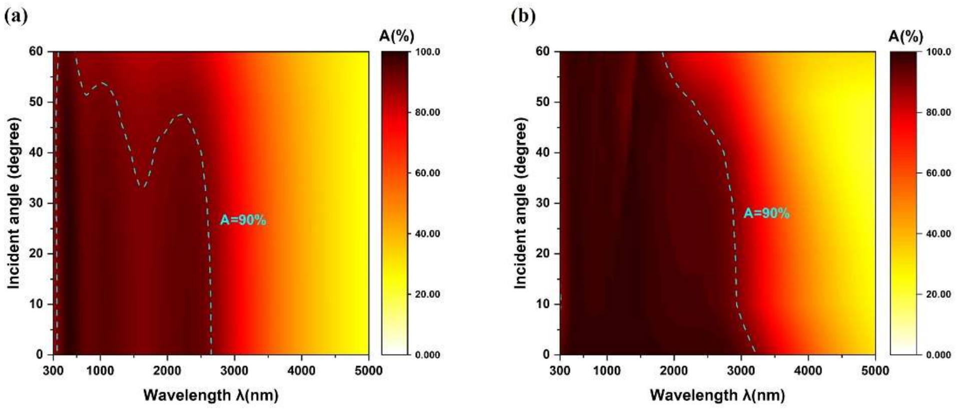

Fig. S4: (a) Simulated absorption spectrum at different incident angles varying from 0° to 60° in 10° steps for unpolarized light of planar multi-cermet absorber. (b) Simulated absorption spectrum at different incident angles varying from 0° to 60° in 10° steps for unpolarized light of nano-cone metamaterial absorber.

5 Effect of nanoparticles on the absorption performance

For convenience of calculations, the surface with nanoparticles is simplified to rough surface in the simulation. We have calculated the absorbance of planar multi-cermet absorber with rough surface and compare it with the smooth one to illustrate the effect of nanoparticles on the absorption performance as Fig.S5 reveals. The average absorbance from 300 to 3000 nm of the absorber with rough surface and smooth surface are 93.5% and 92.5%, respectively. This indicates that the existence of nanoparticles can improve the absorption performance to a certain extent.

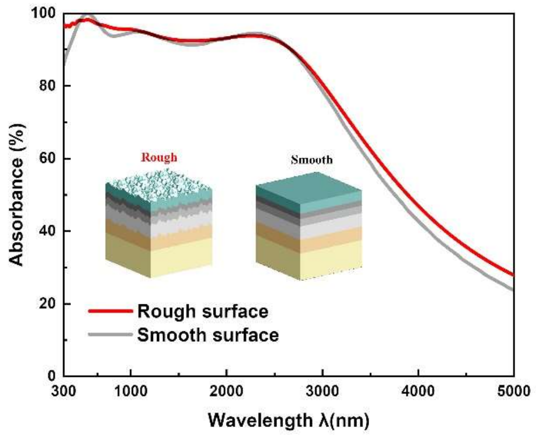

Fig. S5: Simulated absorbance of planar multi-cermet absorber with rough surface and smooth surface.

6 Detailed information of bending test and heating test

It is represented in Fig.S6(a) that the surface morphology of the absorber barely changes after annealing test. After bending test, a narrow crease can be seen in the bend area as shown in Fig.S6(b). However, relative to the surface of the whole absorber, this crease is too small to be ignored. Thus, there is almost no difference in the absorption performance of the sample after annealing and bending test.

(a) (b)

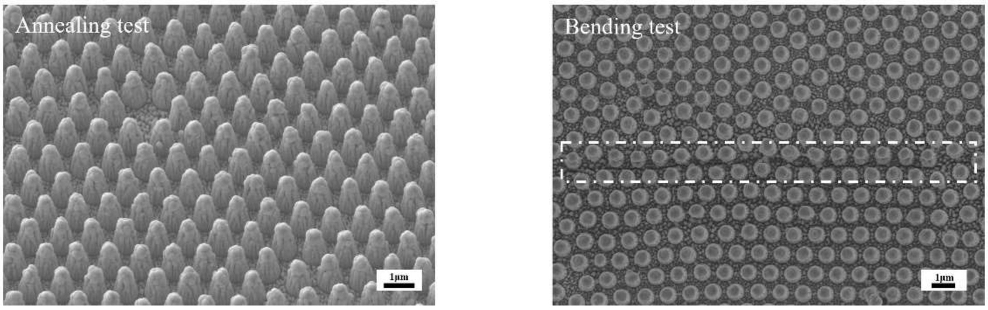

Fig. S6: SEM image of surface morphology of (a) heated sample as well as (b) bent sample.

Bibliography

[1] Macleod, H. A., & Macleod, H. A. (2010). *Thin-film optical filters*. CRC press.
